# *N*-Benzyl-5-(dimethyl­amino)­naphthalene-1-sulfonamide

**DOI:** 10.1107/S1600536811033083

**Published:** 2011-08-27

**Authors:** Pralav Bhatt, Thavendran Govender, Hendrik G. Kruger, Glenn E. M. Maguire

**Affiliations:** a School of Chemistry, University of KwaZulu-Natal, Durban 4000, South Africa; b School of Pharmacy and Pharmacology, University of KwaZulu-Natal, Durban 4000, South Africa

## Abstract

The structure of the title compound, C_19_H_20_N_2_O_2_S, displays inter­molecular N—H⋯O hydrogen bonding, which generates inversion dimers. There is no π–π stacking in the crystal structure. The dihedral angle between the phenyl ring and naphthalene ring system is 59.16 (11)°.

## Related literature

For the use of dansyl fluorescent analogs as insecticides and synergists, see: Himel *et al.* (1971 ▶). Dansyl probes have also been covalently incorporated into a variety of polymeric networks, see: Shea *et al.* (1989 ▶). Dansyl chromophoric compounds have been investigated for intra­molecular energy transfer in aromatic ring systems, see: Schael *et al.* (1998 ▶) and for host–guest inter­ations shown by fluoresence studies of dansyl-labelled calix[6]arene, see: Schonefeld *et al.* (2006 ▶). For related structures, see: Illos *et al.* (2005 ▶); Hongmei *et al.* (2009 ▶); Hong-Wei *et al.* (2009 ▶); Chui *et al.* (2010 ▶).
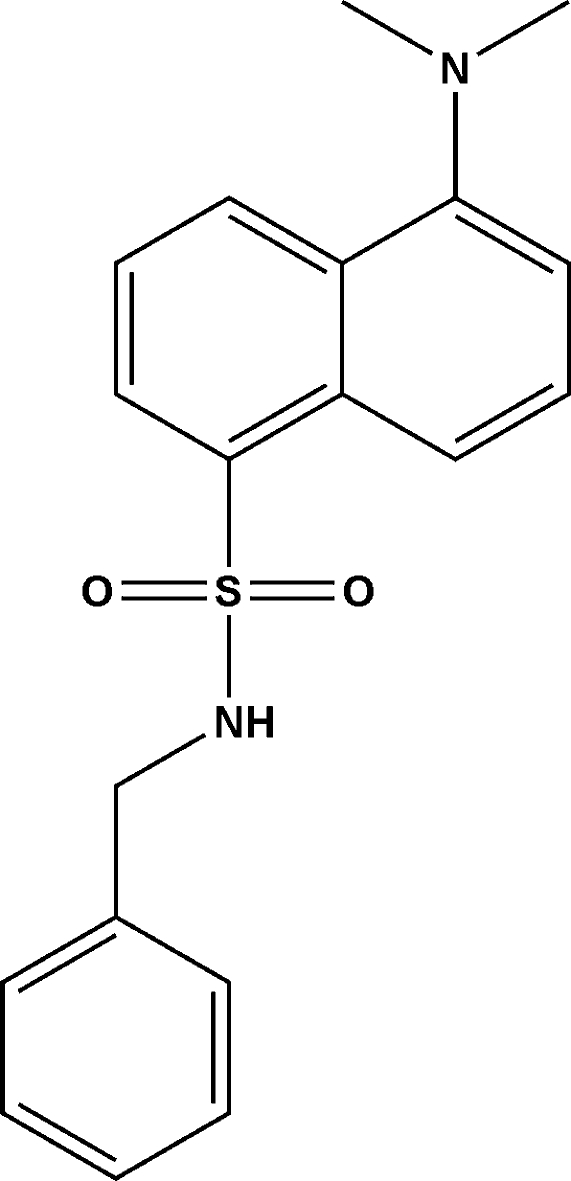


## Experimental

### 

#### Crystal data

C_19_H_20_N_2_O_2_S
*M* *_r_* = 340.43
Monoclinic, 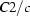

*a* = 16.6635 (5) Å
*b* = 9.5722 (2) Å
*c* = 22.8942 (7) Å
β = 108.779 (1)°
*V* = 3457.38 (16) Å^3^
*Z* = 8
Mo *K*α radiation
μ = 0.20 mm^−1^
*T* = 173 K
0.30 × 0.24 × 0.22 mm

#### Data collection

Nonius KappaCCD diffractometer
4275 measured reflections
4275 independent reflections
3747 reflections with *I* > 2σ(*I*)

#### Refinement

*R*[*F* ^2^ > 2σ(*F* ^2^)] = 0.036
*wR*(*F* ^2^) = 0.096
*S* = 1.03
4275 reflections
223 parameters
1 restraint
H atoms treated by a mixture of independent and constrained refinement
Δρ_max_ = 0.37 e Å^−3^
Δρ_min_ = −0.40 e Å^−3^

### 

Data collection: *COLLECT* (Nonius, 2000 ▶); cell refinement: *DENZO-SMN* (Otwinowski & Minor, 1997 ▶); data reduction: *DENZO-SMN*; program(s) used to solve structure: *SHELXS97* (Sheldrick, 2008 ▶); program(s) used to refine structure: *SHELXL97* (Sheldrick, 2008 ▶); molecular graphics: *OLEX2* (Dolomanov *et al.*, 2009 ▶); software used to prepare material for publication: *SHELXL97*.

## Supplementary Material

Crystal structure: contains datablock(s) I, global. DOI: 10.1107/S1600536811033083/hg5080sup1.cif

Structure factors: contains datablock(s) I. DOI: 10.1107/S1600536811033083/hg5080Isup2.hkl

Supplementary material file. DOI: 10.1107/S1600536811033083/hg5080Isup3.cml

Additional supplementary materials:  crystallographic information; 3D view; checkCIF report

## Figures and Tables

**Table 1:** Hydrogen-bond geometry (Å, °)

*D*—H⋯*A*	*D*—H	H⋯*A*	*D*⋯*A*	*D*—H⋯*A*
N1—H1⋯O2^i^	0.86 (2)	2.12 (2)	2.9351 (14)	158 (2)

Symmetry code: (i) 
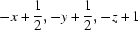
.

**Fig. 1.**
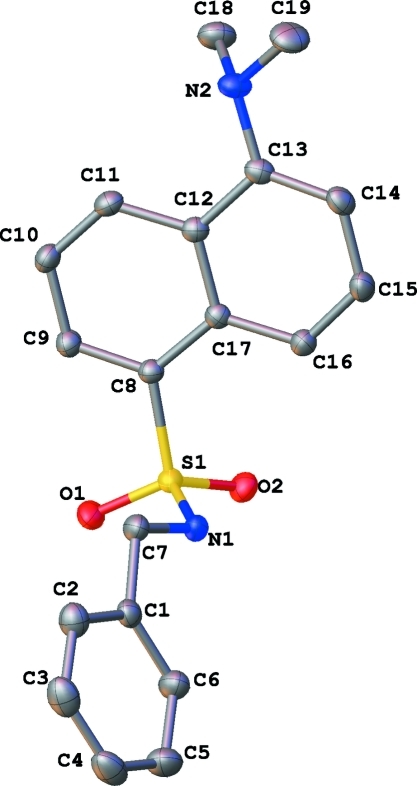
The molecular structure of the title compound with atomic numbering scheme. The H atoms have been omitted for clarity. Displacement elipsoids are drawn at 40% probability.

**Fig. 2.**
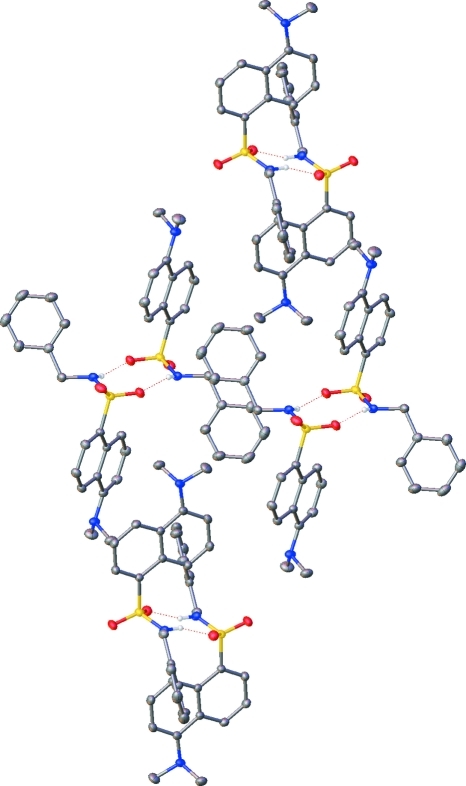
The hydrogen bonding interactions of the title compound along the [110] axis. All H atoms except those involved in hydrogen bonding interactions have been omitted for clarity.

## supplementary crystallographic information

### Comment

Dansyl fluorescent analogs have been reported as insecticides and synergists (Himel *et al.*,1971*).* Dansyl probes have also been covalently incorporated into a variety of polymeric networks (Shea *et al.*,1989). Dansyl chromophoric compounds were investigated for intramolecular energy transfer in aromatic ring systems (Schael *et al.*,1998). Dansyl labelled calix[6]arene are reported to show host–guest interations using fluoresence studies (Schonefeld *et al.*, 2006)*.*

The title compound is a novel benzylated dansyl derivative (Fig. 1.).

There are a number of examples in literature where amino-sulfonamides dansyl structures have shown intermolecular hydrogen bonding. These arrangments can be described in two broad categories. First, where hydrogen bonds occur between the sulfonyl oxygen and the nitrogen of two adjacent molecules in a alternating chain arrangement (Hongmei *et al.*, 2009, Chui *et al.*,2010). Second, where they interact with an adjacent molecule in a head to tail manner, (Illos *et al.*, 2005, Hong-Wei *et al.*, 2009). Our system falls into the latter category. Our structure thus displays N1—H1···O2, 2.9351 (14) Å intermolecular hydrogen bonding, generating inversion dimers (Fig. 2). There is no π–π stacking in the crystal.

### Experimental

To a dry THF 5 ml benzyl amine (107 mg, 1 m*M*) was added triethyl amine (303 mg, 3 m*M*). Dansyl chloride (269 mg, 1 m*M*) was then added and the resulting solution was stirred until the reaction was completed (TLC *R*_f_ = 0.27 in 60% ethyl acetate/hexane). The reaction contents were filtered. The filtrate was evaporated under reduced pressure yielding a yellow oil. To this residue was added 20 ml of dichloromethane and then the organic layer was washed with water and then separated. After drying over anhydrous magnesium sulfate the solvent was evaporated once again under reduced pressure to yield a yellow crystalline solid (240 mg, 71%). *M*.p. = 408 K.

Crystals suitable for X-ray analysis were grown in ethyl acetate/hexane at room temperature.

### Refinement

All non-hydrogen atoms were refined anisotropically. All hydrogen atoms, except H1 on N1, were placed in idealized positions in a riding model and refined with *U*_iso_ set at 1.2 or 1.5 times those of their parent atoms. The position of H1 was located in the difference electron density map and refined with bond length constraint d(N—H) = 0.88 (2) Å.

### Crystal data

**Table:**

C_19_H_20_N_2_O_2_S	*F*(000) = 1440
*M**_r_* = 340.43	*D*_x_ = 1.308 Mg m^−^^3^
Monoclinic, *C*2/*c*	Mo *K*α radiation, λ = 0.71073 Å
Hall symbol: -C 2yc	Cell parameters from 4275 reflections
*a* = 16.6635 (5) Å	θ = 2.5–28.3°
*b* = 9.5722 (2) Å	µ = 0.20 mm^−^^1^
*c* = 22.8942 (7) Å	*T* = 173 K
β = 108.779 (1)°	Block, colourless
*V* = 3457.38 (16) Å^3^	0.30 × 0.24 × 0.22 mm
*Z* = 8	

### Data collection

**Table:**

Nonius KappaCCD diffractometer	3747 reflections with *I* > 2σ(*I*)
Radiation source: fine-focus sealed tube	*R*_int_ = 0.000
graphite	θ_max_ = 28.3°, θ_min_ = 2.5°
1.2° φ scans and ω scans	*h* = 0→22
4275 measured reflections	*k* = 0→12
4275 independent reflections	*l* = −30→28

### Refinement

**Table:**

Refinement on *F*^2^	Primary atom site location: structure-invariant direct methods
Least-squares matrix: full	Secondary atom site location: difference Fourier map
*R*[*F*^2^ > 2σ(*F*^2^)] = 0.036	Hydrogen site location: inferred from neighbouring sites
*wR*(*F*^2^) = 0.096	H atoms treated by a mixture of independent and constrained refinement
*S* = 1.03	*w* = 1/[σ^2^(*F*_o_^2^) + (0.0451*P*)^2^ + 3.0274*P*] where *P* = (*F*_o_^2^ + 2*F*_c_^2^)/3
4275 reflections	(Δ/σ)_max_ < 0.001
223 parameters	Δρ_max_ = 0.37 e Å^−^^3^
1 restraint	Δρ_min_ = −0.40 e Å^−^^3^

### Special details

**Table:**

Experimental. Half sphere of data collected using *COLLECT* strategy (Nonius, 2000). Crystal to detector distance = 40 mm; combination of φ and ω scans of 1.0°, 60 s per °, 2 iterations.
Geometry. All e.s.d.'s (except the e.s.d. in the dihedral angle between two l.s. planes) are estimated using the full covariance matrix. The cell e.s.d.'s are taken into account individually in the estimation of e.s.d.'s in distances, angles and torsion angles; correlations between e.s.d.'s in cell parameters are only used when they are defined by crystal symmetry. An approximate (isotropic) treatment of cell e.s.d.'s is used for estimating e.s.d.'s involving l.s. planes.
Refinement. Refinement of *F*^2^ against ALL reflections. The weighted *R*-factor *wR* and goodness of fit *S* are based on *F*^2^, conventional *R*-factors *R* are based on *F*, with *F* set to zero for negative *F*^2^. The threshold expression of *F*^2^ > σ(*F*^2^) is used only for calculating *R*-factors(gt) *etc*. and is not relevant to the choice of reflections for refinement. *R*-factors based on *F*^2^ are statistically about twice as large as those based on *F*, and *R*- factors based on ALL data will be even larger.

### Fractional atomic coordinates and isotropic or equivalent isotropic displacement parameters (Å^2^)

**Table:**

	*x*	*y*	*z*	*U*_iso_*/*U*_eq_	
S1	0.20046 (2)	0.47743 (3)	0.473136 (13)	0.02444 (9)	
O1	0.18380 (7)	0.60223 (10)	0.50185 (4)	0.0342 (2)	
O2	0.16204 (6)	0.34869 (10)	0.48342 (4)	0.0307 (2)	
N1	0.30155 (7)	0.45093 (11)	0.49739 (5)	0.0276 (2)	
H1	0.3140 (12)	0.3655 (16)	0.4932 (8)	0.050 (5)*	
N2	0.12696 (7)	0.39094 (13)	0.17747 (5)	0.0300 (2)	
C1	0.43601 (8)	0.58023 (13)	0.54635 (6)	0.0268 (3)	
C2	0.49467 (9)	0.68229 (15)	0.54407 (7)	0.0348 (3)	
H2	0.4855	0.7356	0.5075	0.042*	
C3	0.56607 (9)	0.70685 (17)	0.59432 (8)	0.0424 (4)	
H3	0.6051	0.7775	0.5924	0.051*	
C4	0.58056 (10)	0.62813 (18)	0.64761 (8)	0.0435 (4)	
H4	0.6299	0.6438	0.6820	0.052*	
C5	0.52285 (10)	0.52681 (18)	0.65038 (7)	0.0426 (4)	
H5	0.5326	0.4730	0.6869	0.051*	
C6	0.45021 (9)	0.50297 (15)	0.59985 (7)	0.0348 (3)	
H6	0.4106	0.4337	0.6022	0.042*	
C7	0.35869 (8)	0.56091 (13)	0.49002 (6)	0.0286 (3)	
H7A	0.3271	0.6502	0.4809	0.034*	
H7B	0.3774	0.5378	0.4543	0.034*	
C8	0.16952 (7)	0.51250 (12)	0.39278 (5)	0.0221 (2)	
C9	0.14023 (8)	0.64467 (13)	0.37456 (6)	0.0262 (2)	
H9	0.1360	0.7110	0.4043	0.031*	
C10	0.11642 (8)	0.68209 (13)	0.31179 (6)	0.0286 (3)	
H10	0.0963	0.7738	0.2993	0.034*	
C11	0.12215 (8)	0.58726 (13)	0.26895 (6)	0.0263 (3)	
H11	0.1070	0.6146	0.2269	0.032*	
C12	0.15032 (7)	0.44820 (13)	0.28577 (5)	0.0226 (2)	
C13	0.15527 (7)	0.34835 (14)	0.24015 (5)	0.0250 (2)	
C14	0.18974 (8)	0.21871 (14)	0.25901 (6)	0.0295 (3)	
H14	0.1961	0.1540	0.2293	0.035*	
C15	0.21569 (8)	0.18099 (14)	0.32193 (6)	0.0301 (3)	
H15	0.2390	0.0908	0.3339	0.036*	
C16	0.20798 (8)	0.27152 (13)	0.36610 (6)	0.0261 (2)	
H16	0.2239	0.2427	0.4080	0.031*	
C17	0.17616 (7)	0.40859 (12)	0.34938 (5)	0.0217 (2)	
C18	0.03465 (9)	0.40632 (18)	0.15059 (7)	0.0391 (3)	
H18A	0.0086	0.3139	0.1402	0.059*	
H18B	0.0214	0.4634	0.1132	0.059*	
H18C	0.0124	0.4519	0.1805	0.059*	
C19	0.16041 (11)	0.31121 (19)	0.13632 (7)	0.0440 (4)	
H19A	0.2222	0.3041	0.1543	0.066*	
H19B	0.1460	0.3585	0.0963	0.066*	
H19C	0.1356	0.2174	0.1306	0.066*	

### Atomic displacement parameters (Å^2^)

**Table:**

	*U*^11^	*U*^22^	*U*^33^	*U*^12^	*U*^13^	*U*^23^
S1	0.03185 (16)	0.02196 (15)	0.01989 (15)	0.00216 (11)	0.00886 (11)	0.00062 (10)
O1	0.0511 (6)	0.0282 (5)	0.0261 (5)	0.0076 (4)	0.0164 (4)	−0.0023 (4)
O2	0.0370 (5)	0.0270 (5)	0.0301 (5)	−0.0001 (4)	0.0139 (4)	0.0051 (4)
N1	0.0310 (5)	0.0205 (5)	0.0262 (5)	0.0004 (4)	0.0021 (4)	0.0006 (4)
N2	0.0290 (5)	0.0407 (6)	0.0206 (5)	0.0027 (5)	0.0082 (4)	−0.0020 (4)
C1	0.0280 (6)	0.0226 (6)	0.0286 (6)	0.0030 (5)	0.0076 (5)	−0.0041 (5)
C2	0.0314 (7)	0.0320 (7)	0.0420 (8)	0.0005 (5)	0.0130 (6)	−0.0016 (6)
C3	0.0302 (7)	0.0378 (8)	0.0578 (10)	−0.0039 (6)	0.0122 (7)	−0.0103 (7)
C4	0.0307 (7)	0.0461 (9)	0.0453 (9)	0.0016 (6)	0.0003 (6)	−0.0161 (7)
C5	0.0422 (8)	0.0462 (9)	0.0314 (7)	0.0025 (7)	0.0006 (6)	−0.0014 (6)
C6	0.0356 (7)	0.0328 (7)	0.0310 (7)	−0.0022 (6)	0.0039 (6)	0.0001 (5)
C7	0.0338 (6)	0.0233 (6)	0.0262 (6)	−0.0009 (5)	0.0063 (5)	0.0012 (5)
C8	0.0232 (5)	0.0235 (6)	0.0197 (5)	0.0009 (4)	0.0068 (4)	0.0004 (4)
C9	0.0303 (6)	0.0235 (6)	0.0259 (6)	0.0029 (5)	0.0106 (5)	−0.0003 (5)
C10	0.0317 (6)	0.0243 (6)	0.0290 (6)	0.0056 (5)	0.0086 (5)	0.0048 (5)
C11	0.0267 (6)	0.0293 (6)	0.0222 (5)	0.0013 (5)	0.0068 (5)	0.0047 (5)
C12	0.0198 (5)	0.0263 (6)	0.0221 (5)	0.0002 (4)	0.0073 (4)	0.0000 (4)
C13	0.0211 (5)	0.0317 (6)	0.0227 (5)	−0.0004 (5)	0.0076 (4)	−0.0027 (5)
C14	0.0296 (6)	0.0301 (7)	0.0288 (6)	0.0025 (5)	0.0093 (5)	−0.0078 (5)
C15	0.0310 (6)	0.0237 (6)	0.0332 (7)	0.0049 (5)	0.0068 (5)	−0.0023 (5)
C16	0.0270 (6)	0.0240 (6)	0.0252 (6)	0.0018 (5)	0.0055 (5)	0.0003 (5)
C17	0.0193 (5)	0.0232 (6)	0.0224 (5)	−0.0002 (4)	0.0064 (4)	−0.0007 (4)
C18	0.0320 (7)	0.0515 (9)	0.0283 (7)	0.0022 (6)	0.0020 (5)	−0.0016 (6)
C19	0.0523 (9)	0.0567 (10)	0.0288 (7)	0.0067 (8)	0.0212 (7)	−0.0047 (7)

### Geometric parameters (Å, °)

**Table:**

S1—O1	1.4331 (9)	C8—C9	1.3718 (17)
S1—O2	1.4425 (9)	C8—C17	1.4351 (16)
S1—N1	1.6150 (11)	C9—C10	1.4084 (17)
S1—C8	1.7754 (12)	C9—H9	0.9500
N1—C7	1.4651 (17)	C10—C11	1.3622 (18)
N1—H1	0.857 (14)	C10—H10	0.9500
N2—C13	1.4184 (16)	C11—C12	1.4227 (17)
N2—C19	1.4558 (17)	C11—H11	0.9500
N2—C18	1.4687 (17)	C12—C17	1.4306 (16)
C1—C6	1.3843 (19)	C12—C13	1.4375 (16)
C1—C2	1.3948 (19)	C13—C14	1.3771 (19)
C1—C7	1.5125 (17)	C14—C15	1.4113 (18)
C2—C3	1.383 (2)	C14—H14	0.9500
C2—H2	0.9500	C15—C16	1.3687 (18)
C3—C4	1.387 (2)	C15—H15	0.9500
C3—H3	0.9500	C16—C17	1.4207 (17)
C4—C5	1.382 (2)	C16—H16	0.9500
C4—H4	0.9500	C18—H18A	0.9800
C5—C6	1.398 (2)	C18—H18B	0.9800
C5—H5	0.9500	C18—H18C	0.9800
C6—H6	0.9500	C19—H19A	0.9800
C7—H7A	0.9900	C19—H19B	0.9800
C7—H7B	0.9900	C19—H19C	0.9800
			
O1—S1—O2	118.40 (6)	C8—C9—C10	120.04 (11)
O1—S1—N1	107.93 (6)	C8—C9—H9	120.0
O2—S1—N1	106.16 (6)	C10—C9—H9	120.0
O1—S1—C8	106.45 (6)	C11—C10—C9	120.21 (12)
O2—S1—C8	109.54 (6)	C11—C10—H10	119.9
N1—S1—C8	107.99 (6)	C9—C10—H10	119.9
C7—N1—S1	119.48 (9)	C10—C11—C12	121.47 (11)
C7—N1—H1	118.9 (13)	C10—C11—H11	119.3
S1—N1—H1	112.0 (13)	C12—C11—H11	119.3
C13—N2—C19	115.60 (11)	C11—C12—C17	119.24 (11)
C13—N2—C18	114.53 (11)	C11—C12—C13	121.10 (11)
C19—N2—C18	110.42 (11)	C17—C12—C13	119.64 (11)
C6—C1—C2	119.02 (13)	C14—C13—N2	123.05 (11)
C6—C1—C7	123.03 (12)	C14—C13—C12	119.15 (11)
C2—C1—C7	117.94 (12)	N2—C13—C12	117.75 (11)
C3—C2—C1	120.89 (14)	C13—C14—C15	120.69 (11)
C3—C2—H2	119.6	C13—C14—H14	119.7
C1—C2—H2	119.6	C15—C14—H14	119.7
C2—C3—C4	119.91 (14)	C16—C15—C14	121.42 (12)
C2—C3—H3	120.0	C16—C15—H15	119.3
C4—C3—H3	120.0	C14—C15—H15	119.3
C5—C4—C3	119.67 (14)	C15—C16—C17	120.09 (11)
C5—C4—H4	120.2	C15—C16—H16	120.0
C3—C4—H4	120.2	C17—C16—H16	120.0
C4—C5—C6	120.44 (15)	C16—C17—C12	118.87 (11)
C4—C5—H5	119.8	C16—C17—C8	123.98 (11)
C6—C5—H5	119.8	C12—C17—C8	117.14 (11)
C1—C6—C5	120.06 (14)	N2—C18—H18A	109.5
C1—C6—H6	120.0	N2—C18—H18B	109.5
C5—C6—H6	120.0	H18A—C18—H18B	109.5
N1—C7—C1	113.36 (10)	N2—C18—H18C	109.5
N1—C7—H7A	108.9	H18A—C18—H18C	109.5
C1—C7—H7A	108.9	H18B—C18—H18C	109.5
N1—C7—H7B	108.9	N2—C19—H19A	109.5
C1—C7—H7B	108.9	N2—C19—H19B	109.5
H7A—C7—H7B	107.7	H19A—C19—H19B	109.5
C9—C8—C17	121.86 (11)	N2—C19—H19C	109.5
C9—C8—S1	116.47 (9)	H19A—C19—H19C	109.5
C17—C8—S1	121.66 (9)	H19B—C19—H19C	109.5
			
O1—S1—N1—C7	−55.53 (11)	C10—C11—C12—C17	−2.33 (18)
O2—S1—N1—C7	176.58 (9)	C10—C11—C12—C13	179.29 (11)
C8—S1—N1—C7	59.17 (11)	C19—N2—C13—C14	−19.34 (19)
C6—C1—C2—C3	−0.1 (2)	C18—N2—C13—C14	110.75 (14)
C7—C1—C2—C3	178.98 (13)	C19—N2—C13—C12	158.21 (12)
C1—C2—C3—C4	0.8 (2)	C18—N2—C13—C12	−71.70 (15)
C2—C3—C4—C5	−0.9 (2)	C11—C12—C13—C14	174.43 (12)
C3—C4—C5—C6	0.2 (2)	C17—C12—C13—C14	−3.94 (17)
C2—C1—C6—C5	−0.6 (2)	C11—C12—C13—N2	−3.22 (17)
C7—C1—C6—C5	−179.62 (13)	C17—C12—C13—N2	178.41 (10)
C4—C5—C6—C1	0.5 (2)	N2—C13—C14—C15	−179.01 (12)
S1—N1—C7—C1	137.24 (10)	C12—C13—C14—C15	3.47 (19)
C6—C1—C7—N1	−1.11 (18)	C13—C14—C15—C16	−0.4 (2)
C2—C1—C7—N1	179.83 (11)	C14—C15—C16—C17	−2.3 (2)
O1—S1—C8—C9	2.04 (12)	C15—C16—C17—C12	1.77 (18)
O2—S1—C8—C9	131.16 (10)	C15—C16—C17—C8	−177.02 (12)
N1—S1—C8—C9	−113.64 (10)	C11—C12—C17—C16	−177.06 (11)
O1—S1—C8—C17	−178.94 (10)	C13—C12—C17—C16	1.34 (16)
O2—S1—C8—C17	−49.82 (11)	C11—C12—C17—C8	1.81 (16)
N1—S1—C8—C17	65.38 (11)	C13—C12—C17—C8	−179.79 (10)
C17—C8—C9—C10	−0.62 (19)	C9—C8—C17—C16	178.42 (12)
S1—C8—C9—C10	178.40 (10)	S1—C8—C17—C16	−0.55 (16)
C8—C9—C10—C11	0.16 (19)	C9—C8—C17—C12	−0.39 (17)
C9—C10—C11—C12	1.33 (19)	S1—C8—C17—C12	−179.35 (9)

### Hydrogen-bond geometry (Å, °)

**Table:**

*D*—H···*A*	*D*—H	H···*A*	*D*···*A*	*D*—H···*A*
N1—H1···O2^i^	0.86 (2)	2.12 (2)	2.9351 (14)	158.(2)

Symmetry codes: (i) −*x*+1/2, −*y*+1/2, −*z*+1.
